# Advanced *BrainAGE* in older adults with type 2 diabetes mellitus

**DOI:** 10.3389/fnagi.2013.00090

**Published:** 2013-12-17

**Authors:** Katja Franke, Christian Gaser, Brad Manor, Vera Novak

**Affiliations:** ^1^Structural Brain Mapping Group, Department of Psychiatry, Jena University HospitalJena, Germany; ^2^Department of Neurology, Jena University HospitalJena, Germany; ^3^Institute for Aging Research, Hebrew SeniorLifeBoston, MA, USA; ^4^Division of Gerontology, Beth Israel Deaconess Medical Center, Harvard Medical SchoolBoston, MA, USA; ^5^Department of Neurology, Stroke Division, Harvard Medical SchoolBoston, MA, USA

**Keywords:** aging, *BrainAGE*, diabetes mellitus (DM), magnetic resonance imaging (MRI), voxel-based morphometry (VBM)

## Abstract

Aging alters brain structure and function and diabetes mellitus (DM) may accelerate this process. This study investigated the effects of type 2 DM on individual brain aging as well as the relationships between individual brain aging, risk factors, and functional measures. To differentiate a pattern of brain atrophy that deviates from normal brain aging, we used the novel *BrainAGE* approach, which determines the complex multidimensional aging pattern within the whole brain by applying established kernel regression methods to anatomical brain magnetic resonance images (MRI). The “Brain Age Gap Estimation” *(BrainAGE)* score was then calculated as the difference between chronological age and estimated brain age. 185 subjects (98 with type 2 DM) completed an MRI at 3Tesla, laboratory and clinical assessments. Twenty-five subjects (12 with type 2 DM) also completed a follow-up visit after 3.8 ± 1.5 years. The estimated brain age of DM subjects was 4.6 ± 7.2 years greater than their chronological age (*p* = 0.0001), whereas within the control group, estimated brain age was similar to chronological age. As compared to baseline, the average *BrainAGE* scores of DM subjects increased by 0.2 years per follow-up year (*p* = 0.034), whereas the *BrainAGE* scores of controls did not change between baseline and follow-up. At baseline, across all subjects, higher *BrainAGE* scores were associated with greater smoking and alcohol consumption, higher tumor necrosis factor alpha (TNFα) levels, lower verbal fluency scores and more severe deprepession. Within the DM group, higher *BrainAGE* scores were associated with longer diabetes duration (*r* = 0.31, *p* = 0.019) and increased fasting blood glucose levels (*r* = 0.34, *p* = 0.025). In conclusion, type 2 DM is independently associated with structural changes in the brain that reflect advanced aging. The *BrainAGE* approach may thus serve as a clinically relevant biomarker for the detection of abnormal patterns of brain aging associated with type 2 DM.

## Introduction

The global prevalence of type 2 diabetes mellitus (DM) is projected to rise sharply over the coming decades. Individuals aged 65 years and older have a particularly high risk of developing diabetes complications, due to the combination of both modifiable (i.e., lifestyle), and non-modifiable risk factors (Zimmet et al., 2001). Within this population, type 2 DM has been linked to increased brain atrophy (Araki et al., 1994; Schmidt et al., 2004; Last et al., 2007; De Bresser et al., 2010; Van Elderen et al., 2010; Novak et al., 2011), impaired cognitive function (Reijmer et al., 2011), increased risk of depression (Anderson et al., 2001; Ali et al., 2006) and dementia, including both vascular dementia and Alzheimer's disease (AD) (Janson et al., 2004; Xu et al., 2004; Biessels et al., 2006; Velayudhan et al., 2010; Tan et al., 2011; Cheng et al., 2012).

Chronic hyperglycemia is associated with vascular disease and neurotoxicity leading to neuronal damage (Tomlinson and Gardiner, 2008). Within the brain, hyperglycemia appears to induce structural abnormalities resembling the progressive, widespread atrophy often associated with biological aging (Gispen and Biessels, 2000; Biessels et al., 2006). Moreover, within the DM population, such generalized atrophy may be detected at an earlier age (Araki et al., 1994). Clinical manifestations of DM-related brain abnormalities include worse functional status (Stewart and Liolitsa, 1999; Biessels et al., 2006), deficits in cognition [i.e., verbal memory, mental flexibility, and processing speed (Gispen and Biessels, 2000; Cheng et al., 2012)], and depression (Heuser, 2002; Wolkowitz et al., 2010, 2011; Katon et al., 2012). As such, recognition and quantification of subtle deviations from aging-related brain atrophy may afford prospective identification and subsequent treatment of patients with DM who are at risk for clinically-significant functional decline.

**Figure 1:**
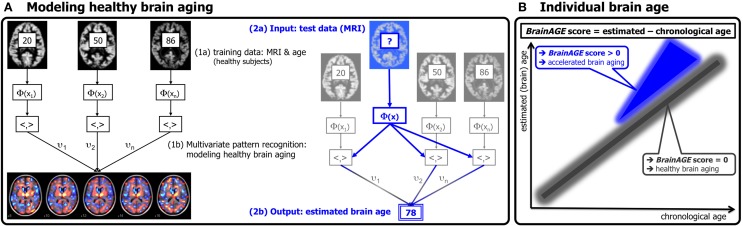
**Depiction of the *BrainAGE* concept. (A)** The model of healthy brain aging is trained with the chronological age and preprocessed structural MRI data of a training sample (left; with an exemplary illustration of the most important voxel locations that were used by the age regression model). Subsequently, the individual brain ages of previously unseen test subjects are estimated, based on their MRI data [blue; picture modified from Schölkopf and Smola (2002)]. **(B)** The difference between the estimated and chronological age results in the *BrainAGE* score. Consequently, positive *BrainAGE* scores indicate accelerated brain aging. [Image reproduced from Franke et al. (2012a), with permission from Hogrefe Publishing, Bern]?, unknown age.

Based on the widespread but well-ordered brain tissue loss that occurs with healthy aging into senescence (Good et al., 2001), we previously proposed a modeling approach to identify abnormal aging-related brain atrophy that may precede the onset of clinical symptoms. We introduced a novel *BrainAGE* approach (Franke et al., 2010, 2012b) based on a database of single time-point structural magnetic resonance imaging (MRI) data that aggregates the complex, multidimensional aging patterns across the whole brain to one single value, i.e., the estimated brain age (Figure 1A). Consequently, subtle deviations in “normal” brain atrophy can be directly quantified in terms of years by analyzing only one standard MRI scan per subject (Figure 1B). Recently, we demonstrated that the *BrainAGE* approach enables the identification of advanced brain aging in subjects with mild cognitive impairment and AD, and observed profound relationships between *BrainAGE*, disease severity, prospective worsening of cognitive functions (Franke et al., 2012a), conversion to AD (Gaser et al., 2013), as well as certain health and lifestyle markers (e.g., the metabolic syndrome; Franke et al., 2013).

In this study, we implemented the *BrainAGE* method to quantify the effects of type 2 DM on individual brain aging in non-demented older adults. We further explored the relationships between individual brain aging and clinically significant lifestyle risk factors (i.e., smoking duration, alcohol intake), clinical laboratory data [i.e., fasting blood glucose level as a potential indicator of hyperglycemia, tumor necrosis factor alpha (TNFα) as a potential indicator of persistent inflammation], and common clinical outcomes (i.e., cognition, depression). We hypothesized that type 2 DM is associated with greater *BrainAGE* scores, and that clinically significant risk factors additionally contribute to this process. We also hypothesized that those individuals with greater *BrainAGE* scores would also exhibit worse outcomes related to cognition and depression.

## Research design and methods

### Subjects

To train the age estimation framework, we used MRI data of 561 healthy subjects [250 males] from the publicly accessible IXI cohort (http://www.brain-development.org; data downloaded in September 2011) aged 20–86 years [mean (SD) = 48.6 (16.5) years; for more sample details see Franke et al. (2010)].

The current *BrainAGE* analyses were conducted using existing records of 185 subjects (98 with diagnosed type 2 DM; Table 1) who previously participated in studies within the Syncope and Falls in the Elderly (SAFE) Laboratory at the Beth Israel Deaconess Medical Center (BIDMC). A subset of these subjects (*n* = 25, 12 with type 2 DM; Table 2) also completed a follow-up MR scan after an average of 3.8 years (*SD* = 1.5).

**Table 1:** **Demographic and clinical variables of the cross-sectional control and type 2 DM groups**.

	**Control group**	**Type 2 DM group**	***p***
No. subjects	87	98	NS
Gender (men/women)	41/46	53/45	NS
Age mean (years)	65.3 (8.5)	64.6 (8.1)	NS
Hypertension (yes/no)	22/65	56/42	<0.05
Diabetes duration (years)	–	11.3 (9.3)	–
GM volume (ml)	528.9 (63.5)	519.0 (52.3)	NS
WM volume (ml)	540.2 (78.3)	536.2 (90.8)	NS
Total brain volume (ml)	1347.7 (147.2)	1338.1 (146.2)	NS
BMI (kg/m^2^)	25.4 (3.7)	28.8 (4.8)	<0.0001
Smoking duration (years)	9.4 (15.1)	10.9 (14.6)	NS
Alcohol intake (dose/ week)	2.0 (3.3)	5.1 (14.3)	NS
Non-fasting blood glucose	82.0 (13.2)	124.0 (56.4)	<0.0001
Fasting blood glucose (Visit 2)	86.7 (13.6)	110.6 (32.4)	NS
TNFα	1.6 (0.7)	1.6 (0.5)	NS
Verbal fluency (T-score)	50.0 (10.1)	39.5 (12.8)	<0.0001
Geriatric depression scale (total score)	3.8 (4.8)	6.4 (6.4)	NS

Data are means ± SD unless otherwise indicated. p denotes between-group comparisons.

**Table 2:**
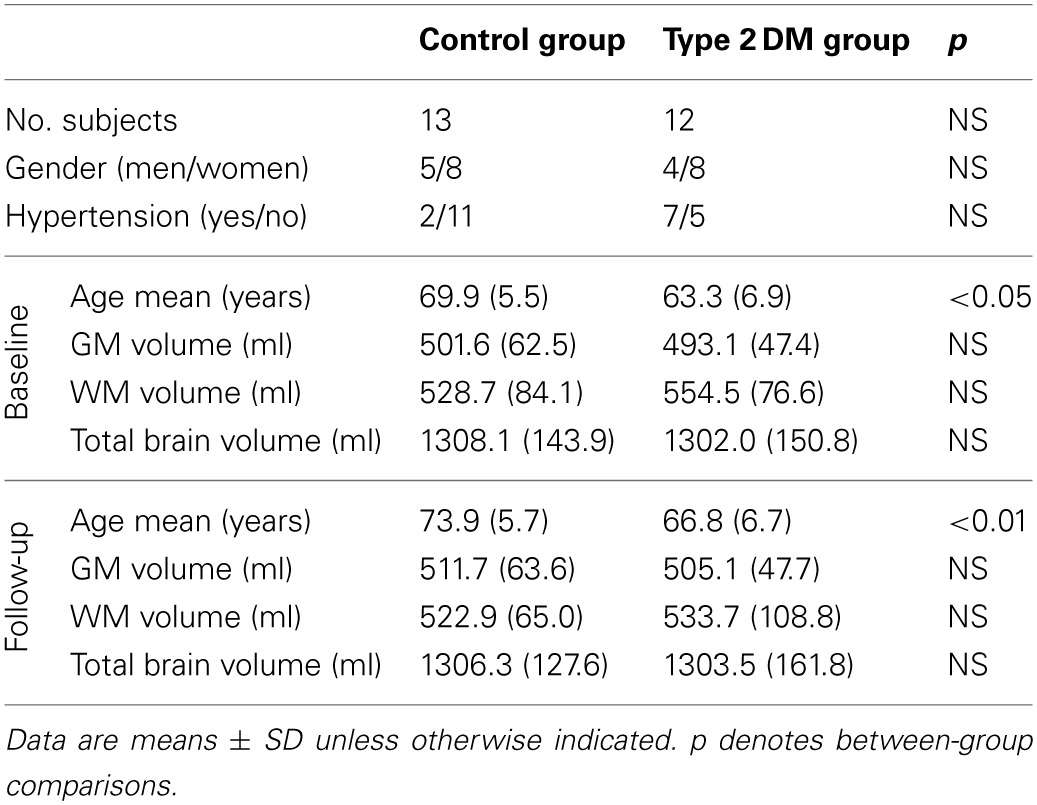
**Demographic and clinical variables of the *longitudinal subsample***.

Data are means ± SD unless otherwise indicated. p denotes between-group comparisons.

Participants were recruited consecutively via advertisement in the local community and provided informed consent as approved by the Institutional Review Board. Controls were required to have normal fasting glucose, but had a similar distribution of risk factors. All participants were screened with a medical history and physical and laboratory examinations. Participants with DM were treated with insulin, oral glucose-control agents (sulfonylurea, second generation agents or their combinations), or diet only. Several participants in each group were treated for hypertension and/or hypercholesterolemia. Excluded were participants with type 1 DM, a history of stroke, myocardial infarction within 6 months, and other clinically important cardiac diseases, arrhythmias, significant nephropathy, kidney or liver transplant, renal or congestive heart failure, carotid artery stenosis (over 50% by medical history and MR angiography), neurological or other systemic disorders; claustrophobia, metal implants, pacemakers, arterial stents incompatible with 3Tesla MRI. All participants were admitted to the Clinical Research Center for an overnight stay. Laboratory chemistries were collected after overnight fasting, and MRI was done before noon. Functional clinical outcomes were acquired through a battery of neuropsychological tests, including assessments for learning and memory, depression, and physical function.

In order to quantify the relationship between *BrainAGE* scores, life-style risk factors and clinical outcomes, the following data were extracted: body mass index (BMI), smoking duration, alcohol intake, non-fasting blood glucose levels, parameters of diabetes control (duration, fasting blood glucose levels), common clinical outcomes [i.e., verbal fluency, more specifically “semantic fluency,” requiring the generation of exemplars of the category “animals” (Harrison et al., 2000; Fisher et al., 2004) and depression as measured with the Geriatric Depression Scale (GDS; Yesavage, 1988)], and inflammation markers (TNFα).

### Magnetic resonance imaging

All studies were performed within the Center for Advanced MR Imaging at the BIDMC on the same 3Tesla GE HDx MRI scanner using a quadrature and phase array head coils (GE Medical Systems, Milwaukee, WI). Anatomical images were acquired using 3-D magnetization prepared rapid gradient echo (MP-RAGE) (*T*_*R*_/*T*_*E*_/*T*_*I*_ = 7.8/3.1/600 ms, 3.0 mm slice thickness, 52 slices, bandwidth = 122 Hz per pixel, flip angle = 10°, 24 cm × 24 cm FOV, 256 × 192 matrix size) and fluid attenuated inversion recovery (FLAIR) (*T*_*R*_/*T*_*E*_/*T*_*I*_ = 11000/161/2250 ms, 5 mm slice thickness, 30 slices, bandwidth = 122 Hz per pixel, flip angle = 90°, 24 cm × 24 cm FOV, 256 × 160 matrix size) sequences.

### Preprocessing of MRI data and data reduction

Preprocessing of the T1-weighted images was done using the SPM8 package (http://www.fil.ion.ucl.ac.uk/spm) and the VBM8 toolbox (http://dbm.neuro.uni-jena.de), running under MATLAB. All T1-weighted images were corrected for bias-field inhomogeneities, then spatially normalized and segmented into gray matter (GM), white matter (WM), and cerebrospinal fluid (CSF) within the same generative model (Ashburner and Friston, 2005). The segmentation procedure was extended by accounting for partial volume effects (Tohka et al., 2004), by applying adaptive maximum a posteriori estimations (Rajapakse et al., 1997), and by using a hidden Markov random field model (Cuadra et al., 2005; Gaser, 2009). The images were processed with affine registration and smoothed with 8-mm full-width-at-half-maximum smoothing kernels. Spatial resolution was set to 8 mm. For further data reduction, principal component analysis (PCA) was performed on the training sample with subsequently applying the estimated transformation parameters to the test sample. PCA was done using the “MATLAB Toolbox for Dimensionality Reduction” (http://homepage.tudelft.nl/19j49/Matlab_Toolbox_for_Dimensionality_Reduction.html), running under MATLAB.

### Age estimation framework

The *BrainAGE* framework utilizes a machine-learning pattern recognition method, namely relevance vector regression (RVR; Tipping, 2001). It was recently developed to estimate individual brain ages based on T1-weighted images (Franke et al., 2010). In general, the model is trained with preprocessed whole brain structural MRI data of the training sample (here: the IXI sample). Subsequently, the brain age of each test subject can be estimated using the individual tissue-classified MRI data, aggregating the complex, multidimensional aging pattern across the whole brain into one single value (Figure 1A). The difference between estimated and true chronological age will reveal the individual *brain age gap estimation* (*BrainAGE*) score. Consequently, the *BrainAGE* score directly quantifies the amount of acceleration or deceleration of brain aging. For example, if a 70 years old individual has a *BrainAGE* score of +5 years, this means that this individual shows the typical atrophy pattern of a 75 years old individual (Figure 1B). Recent work has demonstrated that this method provides reliable and stable estimates (Franke et al., 2012a). Specifically, the *BrainAGE* scores calculated from two shortly delayed scans on the same MRI scanner, as well as on separate 1.5T and 3.0T scanners, produced intraclass correlation coefficients (ICC) of 0.93 and 0.90, respectively.

Within this study, the *BrainAGE* framework was applied using the linear combination of preprocessed (as described in the section “Preprocessing of MRI data and data reduction”) GM and WM images. For training the model as well as for predicting individual brain ages, we used “The Spider” (http://www.kyb.mpg.de/bs/people/spider/main.html), a freely available toolbox running under MATLAB. For an illustration of the most important features (i.e., the importance of voxel locations for regression with age) that were used by the RVR to model normal brain aging and more detailed information please refer to Franke et al. (2010).

### Statistical analysis

Descriptive statistics were used to summarize all variables. Demographic and laboratory data were compared between the control and the DM groups using analysis of variance (ANOVA) for continuous variables or Kruskal–Wallis tests for categorical variables and variables that were not normally distributed. Normality was tested using Shapiro–Wilk tests. Cross-sectionally, within-group differences between estimated brain age and chronological age were tested using Student's *t*-test.

The effect of DM on *BrainAGE* was determined with ANOVA. The dependent variable was the *BrainAGE* score. Model effects included group (i.e., DM and non-DM controls), hypertension (i.e., with/without hypertension), and gender.

Relationships between *BrainAGE* and clinical parameters were then analyzed in the whole sample (i.e., DM and non-DM subjects together), controlling for age, gender, and diabetes duration (with diabetes duration = 0 years for non-DM controls). As not all subjects had values for all clinical variables, univariate correlation analyses were used (instead of multivariate models) to assess the relationships between *BrainAGE* and distinguished lifestyle measures (i.e., BMI, smoking duration, alcohol intake), clinical laboratory data (i.e., fasting blood glucose level, TNFα) and functional measures (i.e., T-score for verbal fluency, total GDS score for depression). In order to control for covariates, Pearson's pairwise correlation were used for normally distributed variables, and Spearman's correlations were used for variables that are not normally distributed, with adjustment for age, gender, and diabetes duration (right-tailed for verbal fluency, left-tailed for all others). To control for multiple comparisons, Bonferroni–Holm correction (Holm, 1979) was applied, adjusting the *p*-value for the number of variables analyzed (i.e., 7).

The effect of diabetes-status within the relationships between *BrainAGE* and lifestyle parameters, clinical laboratory data and outcome measures were investigated by performing analysis of covariance (ANCOVA). Each specific ANCOVA included all those subjects who were measured in each specific clinical variable, sub-grouped by DM. Since fasting blood glucose levels were provided for only three non-DM control subjects, this variable was excluded from this analysis. For all other variables, the model fitted separate lines for both groups, thus, allowing the intercept as well as the slopes to vary between both groups.

To further explore the relationship between *BrainAGE* and clinical parameters, the whole sample was divided into quartiles for each of the significantly related lifestyle measures (i.e., smoking duration, alcohol intake), clinical laboratory data (i.e., fasting blood glucose level, TNFα), and outcome measures (i.e., verbal fluency, depression). To illustrate the relationships between individual brain aging and extreme levels in each of these variables, the *BrainAGE* scores in the 1st quartile (lowest 25% of values) of each lifestyle and functionality measure were tested against the *BrainAGE* scores in 4th quartile (highest 25% of values) of each lifestyle and functionality measure, using one-tailed *t*-tests (right-tailed for verbal fluency, left-tailed for all others). Bonferroni–Holm-adjusted *p*-values were used to determine significance.

Within the subsample that completed two MRI scans, the longitudinal changes in individual *BrainAGE* scores were fitted against time between both scans with a multivariate linear regression model. *BrainAGE* scores at baseline and follow-up visit, as well as longitudinal changes in *BrainAGE* were compared between both groups using ANOVA.

The Shapiro–Wilk test was performed using JMP 9.0 (www.jmp.com). All other testing was performed using MATLAB 7.11. (www.mathworks.com).

## Results

### Group characteristics

All variables except diabetes duration, BMI, alcohol intake and GDS scores were normally distributed. Age, gender, GM, WM, and total brain volumes did not differ between groups (Table 1). The DM group had higher BMI (*p* < 0.0001), higher non-fasting blood glucose levels (*p* < 0.0001), greater prevalence of hypertension (*p* < 0.05), and worse performance in verbal fluency (*p* < 0.0001) than controls (Table 1).

### Cross-sectional *BrainAGE* analyses

**Figure 2:**
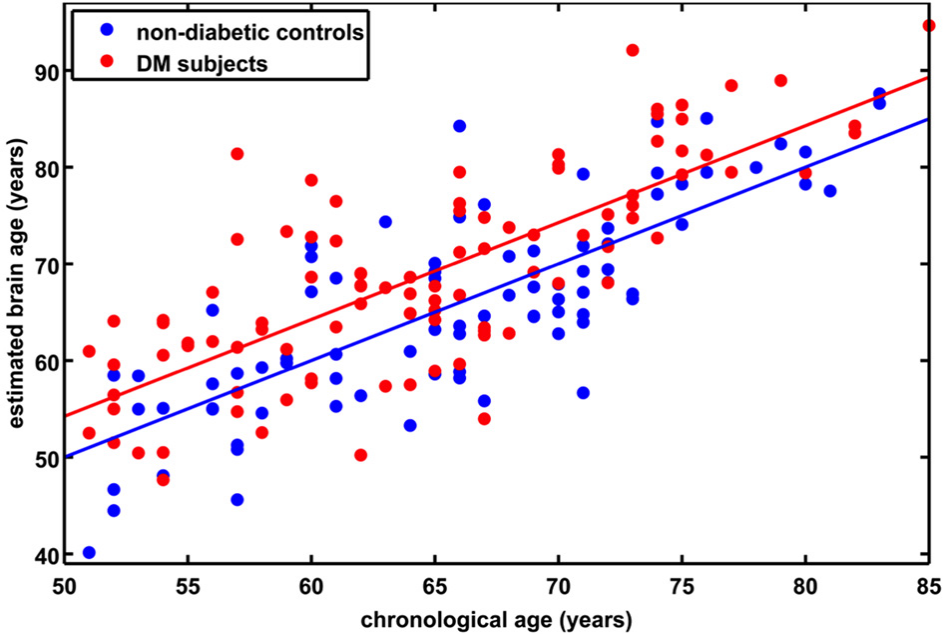
**Estimated brain age vs. chronological age for controls and subjects with type 2 DM**. The *BrainAGE* scores (i.e., the difference between the estimated and the chronological age) differed between groups, with mean (± *SD*) *BrainAGE* scores of 0.0 ± 6.7 years in healthy controls (blue) and 4.6 ± 7.2 years in type 2 DM subjects (red; *p* < 0.0001).

**Figure 3:**
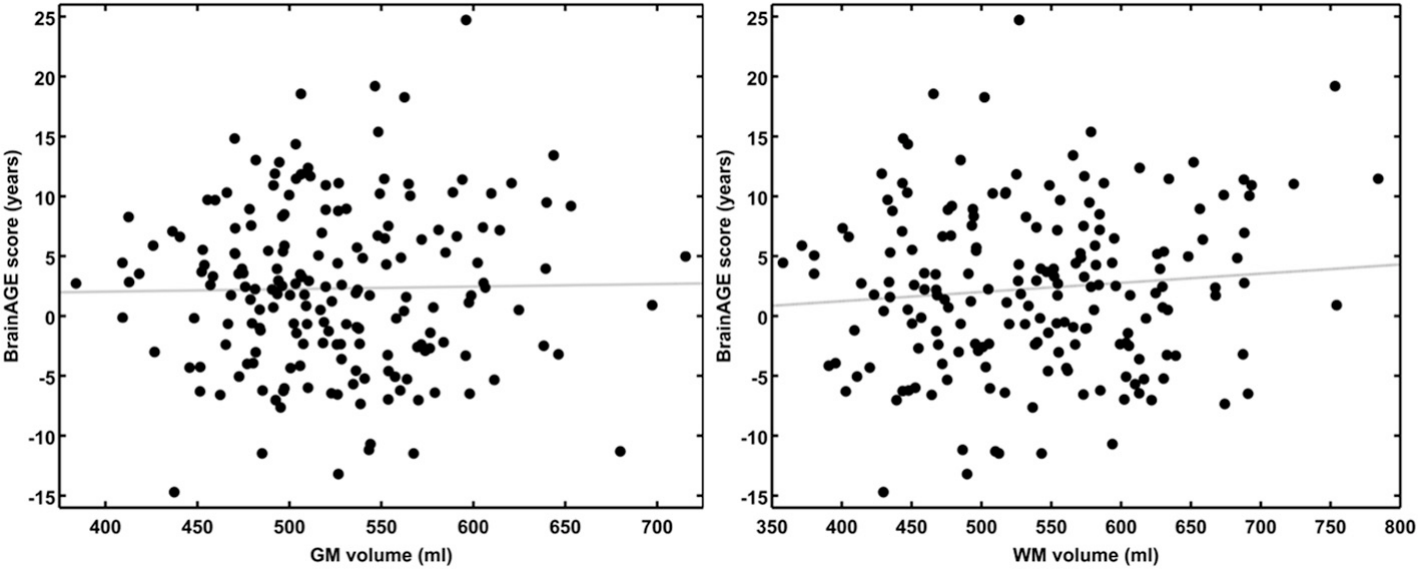
***BrainAGE* scores plotted against GM and WM volumes for all subjects**. *BrainAGE* scores did not correlate to either GM (left; *r* = 0.02, *p* = 0.81) or WM volumes (right; *r* = 0.09, *p* = 0.20).

Although brain volumes did not differ between the groups, the DM subjects had significantly higher *BrainAGE* scores than controls (*F* = 17.2; *p* = 0.0001; Figure 2). Additionally, *BrainAGE* scores did not correlate to brain volumes (Figure 3). Within the control group, estimated brain age was similar to chronological age [*t*_(0.975, 86)_ = 0.0; *p* = 1.0]. In DM subjects, however, the average *BrainAGE* score was 4.6 years (*SD* = 7.2); i.e., their estimated brain age was 4.6 years greater than their chronological age [*t*_(0.975, 97)_ = 6.4; *p* = 0.0001]. Additionally, within the DM group, those with longer diabetes duration had higher *BrainAGE* scores (*r* = 0.31, *p* = 0.019). This relationship was independent of age, gender, and duration of hypertension history.

Across all subjects, *BrainAGE* scores were higher in males as compared to females (*F* = 7.7; *p* = 0.006). There were no effects for hypertension (*F* = 0.0; *p* = 0.9), or any interaction (group * hypertension: *F* = 0.6; *p* = 0.46; group * gender: *F* = 0.7; *p* = 0.41; hypertension * gender: *F* = 0.1; *p* = 0.79).

Across all subjects, higher *BrainAGE* scores were significantly correlated with lifestyle factors, i.e., increased duration of smoking (*r* = 0.20, *p* = 0.007) and greater alcohol consumption (*r* = 0.24, *p* = 0.001), as well as clinical laboratory data, i.e., higher fasting blood glucose (*r* = 0.34, *p* = 0.025) and TNFα (*r* = 0.29, *p* = 0.01) levels. Higher *BrainAGE* scores were also correlated with lower verbal fluency (*r* = −0.25, *p* = 0.006) and higher depression scores (*r* = 0.23, *p* = 0.012). All correlations were independent of age, gender, and diabetes duration.

**Figure 4:**
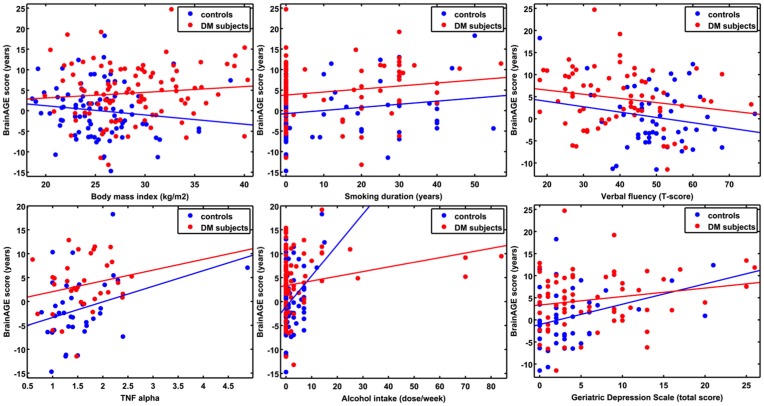
**ANCOVA plots for *BrainAGE* scores and distinguished variables**. *BrainAGE* scores are plotted against BMI, smoking duration, alcohol intake, TNFα, verbal fluency, and GDS scores for non-DM controls (blue) and subjects with type 2 DM (red). In both groups, higher *BrainAGE* scores were significantly related to increased smoking duration (*p* < 0.05), increased alcohol intake (*p* < 0.01), increased TNFα (*p* < 0.05), decreased verbal fluency (*p* < 0.05), and increased GDS scores (*p* < 0.01).

Additionally, ANCOVAs were performed to investigate the effects of DM status on the relationships between *BrainAGE* scores and distinguished lifestyle factors, clinical variables, and outcome measures. Although *BrainAGE* scores were generally higher in DM subjects, higher *BrainAGE* scores were also related to increased smoking duration (*F* = 5.13, *p* < 0.05), increased alcohol intake (*F* = 7.63, *p* < 0.01), increased TNFα (*F* = 6.24, *p* < 0.05), decreased verbal fluency (*F* = 4.07, *p* < 0.05), and increased GDS scores (*F* = 7.17, *p* < 0.01) in DM subjects as well as in non-DM controls (Table 3, Figure 4).

**Table 3:** **ANCOVA results for *BrainAGE* scores and distinguished variables**.

	**Model**						**Coefficient estimates**			
	**Group**		**Variable value**		**Group × Value**		**Intercept**		**Slope**	
	***F***	***p***	***F***	***p***	***F***	***p***	***t***	***p***	***t***	***p***
BMI	17.4	**0.0001**	0.02	0.89	2.4	0.12	0.94	0.35	0.36	0.39
Smoking duration	21.4	**0.0001**	5.13	**0.02**	0.0	0.97	2.6	**0.01**	2.26	**0.02**
Alcohol intake	11.8	**0.0007**	7.63	**0.006**	6.82	**0.009**	2.13	**0.03**	3.54	**0.0005**
TNFα	11.2	**0.001**	6.24	**0.01**	0.16	0.69	1.61	0.11	2.18	**0.03**
Verbal fluency	6.28	**0.01**	4.07	**0.04**	0.06	0.80	2.79	**0.006**	1.96	0.05
GDS	7.12	**0.009**	7.17	**0.008**	1.46	0.23	1.47	0.14	2.94	**0.004**

Bold type = significant test results.

**Figure 5:**
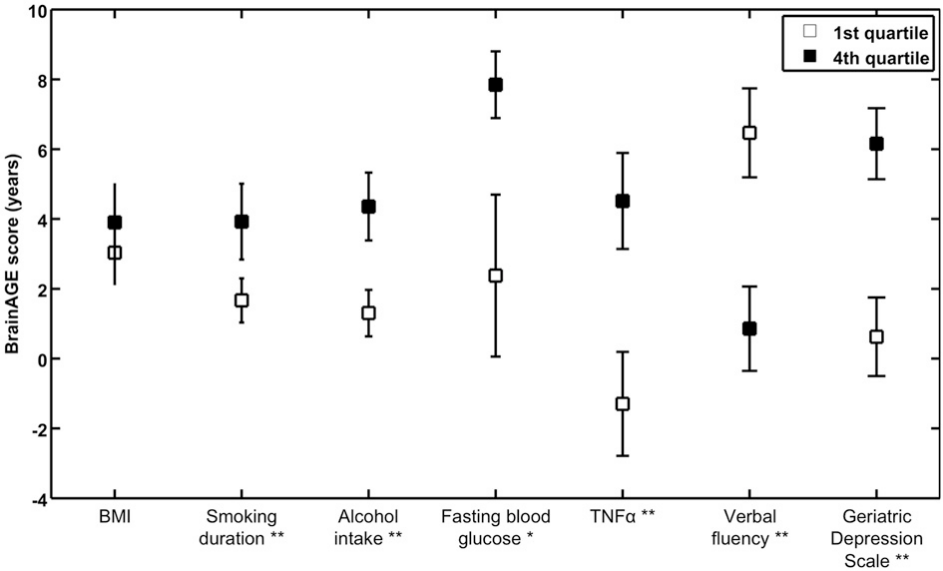
**Quartile analyses**. Mean *BrainAGE* scores in subjects with values in the 1st and 4th quartiles of distinguished variables. Error bars depict the standard error of the mean (SEM). ^*^p < 0.05, ^**^p < 0.01.

To exemplarily quantify the relationship between brain atrophy and lifestyle factors, clinical laboratory data and functionality, the *BrainAGE* scores of subjects with the lowest values in those measures (i.e., 1st quartile) vs. subjects with the highest values in those measures (i.e., 4th quartile) were contrasted (Table 4; Figure 5). These analyses resulted in significant differences in *BrainAGE* of 3.4 years for smoking duration (*p* = 0.004), 4.1 years for alcohol intake (*p* = 0.003), 5.5 years for fasting blood glucose (*p* = 0.02), 5.4 years for TNFα (*p* = 0.006), 5.6 years for verbal fluency (*p* = 0.001), and 5.4 years for depression scores (*p* = 0.002).

**Table 4:** **Comparison of *BrainAGE* scores between the quartile groups in the whole sample**.

	**Mean (*SD*) *BrainAGE* score (years)**				***p* for trend**
	**1st quartile**	**2nd quartile**	**3rd quartile**	**4th quartile**	
BMI	3.04 (6.06)	0.59 (7.54)	1.68 (5.75)	3.90 (7.37)	0.29
Smoking duration	1.67 (6.43)	–	0.87 (6.51)	5.07 (7.25)	**0.012**
Alcohol intake	1.30 (6.57)	–	0.82 (5.97)	5.42 (6.07)	**0.002**
Fasting blood glucose	2.38 (7.34)	0.13 (7.67)	5.21 (4.22)	7.85 (3.02)	**0.036**
TNFα	−1.30 (6.31)	0.20 (6.64)	0.51 (7.16)	4.11 (5.75)	0.10
Verbal fluency	6.47 (6.98)	3.14 (6.92)	0.72 (5.38)	0.86 (6.63)	**0.002**
GDS	0.62 (6.56)	2.78 (7.65)	2.72 (5.37)	6.01 (5.73)	**0.015**

Bold type = significant test results.

### Longitudinal *BrainAGE* analyses

**Figure 6:**
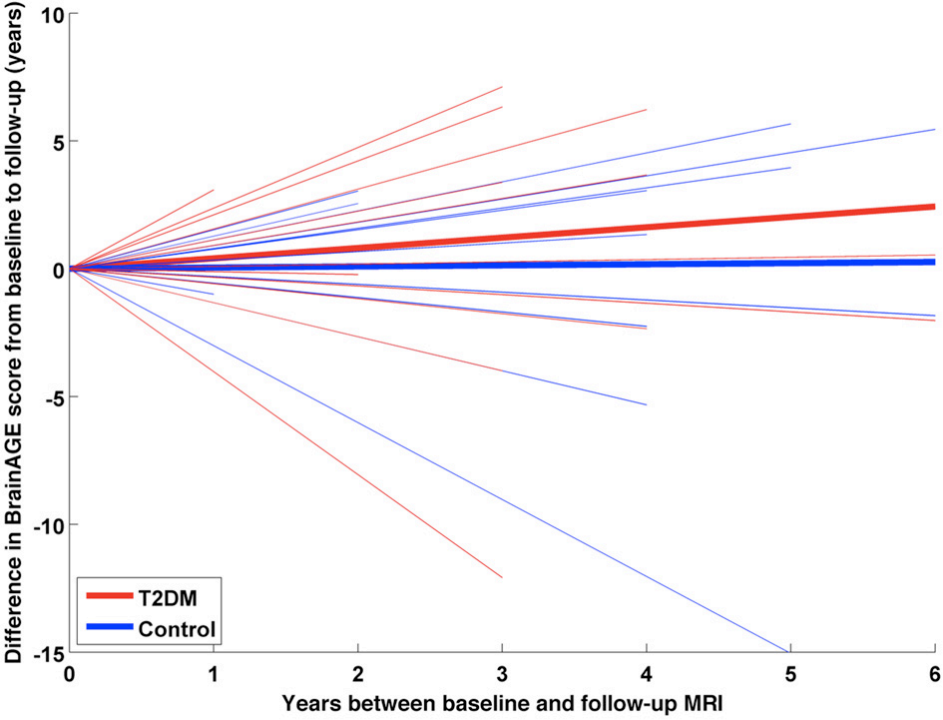
**Longitudinal *BrainAGE* changes in controls and type 2 DM subjects**. Longitudinal changes in *BrainAGE* scores for non-DM control subjects (blue) and type 2 DM subjects (red). Thin lines represent individual changes in *BrainAGE* over time; thick lines indicate estimated average changes for each group. The change in *BrainAGE* over time was dependent upon group (*p* = 0.01), providing preliminary longitudinal evidence that type 2 DM accelerates brain aging.

A subsample of 25 subjects (12 DM subjects and 13 controls) completed a second MRI scan 3.8 ± 1.5 years after their baseline assessment. In this subsample, GM, WM as well as total brain volumes did not differ between groups (Table 2), or across time points (GM volume: *p* = 0.48; WM volume: *p* = 0.58; total brain volume: *p* = 0.99). Interestingly, however, we observed a change in *BrainAGE* over time that was dependent upon group (*F* = 6.9; *p* = 0.015; Figure 6). Specifically, as compared to baseline, average *BrainAGE* scores increased in DM subjects by 0.2 years per follow-up year. Within the control group, as expected, *BrainAGE* scores were similar to chronological age at baseline and follow-up and therefore, did not change over time. In other words, whereas the *BrainAGE* scores of patients with DM were on average 5.1 years higher than controls at baseline (*F* = 6.2; *p* = 0.020), they were on average 5.9 years higher than controls at follow-up (*F* = 5.0; *p* = 0.034).

## Discussion

This study implemented a novel MRI-based biomarker that comprises well-established and fully automated steps for processing standard T1-weighted MR images, aggregating the complex, multidimensional aging pattern across the whole brain into one single value; i.e., the *BrainAGE* score. This method has the advantage of accurately and reliably estimating brain age with minimal preprocessing and parameter optimization (Franke et al., 2010, 2012b), using a single anatomical scan. The *BrainAGE* score directly quantifies subtle deviations from the normal brain-aging pattern and may therefore provide clinically important prognostic information.

In this study, the *BrainAGE* approach was used to determine the effects of type 2 DM on brain aging. Although GM, WM, and total brain volumes did not differ between groups, *BrainAGE* scores were on average 4.6 years greater in DM subjects as compared to non-DM controls. Moreover, *BrainAGE* scores tended to be higher in those with longer diabetes duration and higher fasting blood glucose levels, suggesting a potential link between worse glycemic control and pathologic brain atrophy. Longitudinal analyses further indicated that DM might result in greater increases in *BrainAGE* scores over time (despite no detectable change in global brain tissue volumetrics). Together, these results suggest that the *BrainAGE* score may be sensitive to subtle, glucose-mediated structural brain changes that reflect a pattern of premature brain aging (Araki et al., 1994; Gispen and Biessels, 2000; Biessels et al., 2006; Van Elderen et al., 2010; Velayudhan et al., 2010; Tan et al., 2011).

This study also revealed that individual brain aging was correlated with numerous clinical outcomes. Across all subjects, and independently of diabetes duration, age, and gender, those with higher *BrainAGE* scores consumed more alcohol. This observation is supported by recent studies suggesting a *U*-shaped relationship between alcohol consumption and cognitive impairment (Anttila et al., 2004; Solfrizzi et al., 2008). Higher *BrainAGE* scores were also linked to increased TNFα levels, which are now believed to play a central role in the pathogenesis of AD (Tobinick and Gross, 2008). To this end, those with higher *BrainAGE* scores also tended to have worse verbal fluency. Finally, those subjects with higher *BrainAGE* scores were more likely to have more severe depressive symptoms, which is in line with recent studies linking depression to both advanced brain aging (Heuser, 2002; Wolkowitz et al., 2010, 2011) as increased risk of dementia (Katon et al., 2012).

The *BrainAGE* approach was designed to recognize and indicate deviations in age-related spatiotemporal brain changes. Subjects with a high *BrainAGE* score may thus be at risk for several neurodegenerative diseases and related functional declines. Higher *BrainAGE* scores as well as profound correlations to disease severity and prospective worsening of cognitive functions have already been observed in subjects with mild cognitive impairment and AD (Franke et al., 2012a). The *BrainAGE* approach was even capable of identifying subjects who will be diagnosed with AD up to three years in advance, with each additional year in the *BrainAGE* score being associated with a 10% greater risk of developing AD (Gaser et al., 2013). As such, larger prospective trials are warranted to confirm our initial observation that type 2 DM leads to premature brain aging, and to determine whether this pattern is similar to those of other neurodegenerative diseases. In future research, we aim to further explore and disentangle age- and unrelated disease-based processes of brain atrophy in neurodegenerative diseases (e.g., vascular dementia, AD) as well as its effects on *BrainAGE* estimations.

In the present study, there was considerable variance associated with individual *BrainAGE* scores, as well as intra-individual changes in *BrainAGE* scores over time. As we have previously reported (Franke et al., 2013), and confirmed in this study, a number of nutrition, lifestyle, and health parameters likely contribute to this variance. For example, in older male adults without major disease, 39% of the inter-subject variance in *BrainAGE* was explained by the set of clinical markers under consideration, with markers of the metabolic syndrome mainly contributing to this variance (Franke et al., 2013). As individual changes in lifestyle (e.g., smoking cessation, physical activity, intake of unsaturated fatty acids, moderate alcohol intake) were shown to lower the risk of cognitive decline and dementia (Erickson et al., 2010; Frisardi et al., 2010; Nepal et al., 2010), such lifestyle changes may be also related to a decrease in individual *BrainAGE*. Future research is therefore warranted to determine the effects of individual health and lifestyle modification, as well as improved DM control (e.g., a lowering of blood glucose levels), on longitudinal changes in individual *BrainAGE* scores.

It is of note that WM lesions, which occur primarily due to cerebro-vascular diseases (Hadjidemetriou et al., 2008; Zhan et al., 2009), are not detected in the segmentation approach used within the *BrainAGE* analysis. Such lesions segmented as GM may therefore influence the RVR. However, as the prevalence of WM lesions was minimal in the current cohort, it is unlikely that this limitation influenced the training of “normal brain aging.” Thus, even though the current *BrainAGE* method has high test-retest reliability (Franke et al., 2012a), it may benefit from the development and implementation of segmentation methods that enable automated detection of WM lesions even without any additional FLAIR sequence (Klöppel et al., 2011).

As not all subjects had values for all clinical variables, we were unable to utilize multivariate models to examine the relationship between *BrainAGE* and health-related outcomes, as this approach would have resulted in an extreme reduction in sample size (*n* = 17). Future studies with larger samples are therefore needed to enable multivariate analyses designed to identify the complex interactions between brain aging, lifestyle factors, and clinical outcomes. Moreover, as our prospective cohort was rather small, it still remains unclear whether the presence of type 2 DM and/or lifestyle risk factors represents the cause or consequence of observed associations. Further research is therefore needed to extend our results on the longitudinal relationships between individual brain aging and miscellaneous risk factors (e.g., diabetes, lifestyle, depression) in a larger population-based sample. Furthermore, the relationship between the duration of exposure to risk factors and accelerated brain aging, and whether reversal of modifiable factors might decelerate the progression of brain aging, should be explored.

As *BrainAGE* scores are calculated from a single T1-weighted MRI per subject, using processing techniques that can be fully automated with multi-center data, this approach may be easily implemented into clinical practice in order to encourage the identification of subtle, yet clinically-significant, changes in brain structure. With regards to type 2 DM, the implications of this study may lead to a clinical tool that identifies people at risk of faster degradation of brain structure and function and potential risk for dementias, thus, contributing to an early diagnosis of neurodegenerative diseases and facilitating early treatment or preventative interventions.

## Author contributions

Katja Franke is the guarantor of this work and, as such, had full access to all the data in the study and takes responsibility for the integrity of the data and the accuracy of the data analysis. Katja Franke analyzed data, wrote the manuscript, and edited the manuscript. Christian Gaser contributed to discussion and reviewed manuscript. Brad Manor researched data, contributed to discussion and reviewed/edited manuscript. Vera Novak researched data, contributed to discussion and reviewed/edited manuscript.

### Conflict of interest statement

The authors declare that the research was conducted in the absence of any commercial or financial relationships that could be construed as a potential conflict of interest.
